# Malian adults maintain serologic responses to virulent PfEMP1s amid seasonal patterns of fluctuation

**DOI:** 10.1038/s41598-021-92974-7

**Published:** 2021-07-13

**Authors:** Noah T. Ventimiglia, Emily M. Stucke, Drissa Coulibaly, Andrea A. Berry, Kirsten E. Lyke, Matthew B. Laurens, Jason A. Bailey, Matthew Adams, Amadou Niangaly, Abdoulaye K. Kone, Shannon Takala-Harrison, Bourema Kouriba, Ogobara K. Doumbo, Phillip L. Felgner, Christopher V. Plowe, Mahamadou A. Thera, Mark A. Travassos

**Affiliations:** 1 University of Maryland School of Medicine, Baltimore, MD USA; 2 University of Sciences, Techniques and Technologies, Bamako, Mali; 3 University of California, Irvine, CA USA

**Keywords:** Parasite host response, Malaria, Malaria

## Abstract

*Plasmodium falciparum* erythrocyte membrane protein-1s (PfEMP1s), diverse malaria proteins expressed on the infected erythrocyte surface, play an important role in pathogenesis, mediating adhesion to host vascular endothelium. Antibodies to particular non-CD36-binding PfEMP1s are associated with protection against severe disease. We hypothesized that given lifelong *P. falciparum* exposure, Malian adults would have broad PfEMP1 serorecognition and high seroreactivity levels during follow-up, particularly to non-CD36-binding PfEMP1s such as those that attach to endothelial protein C receptor (EPCR) and intercellular adhesion molecule-1 (ICAM-1). Using a protein microarray, we determined serologic responses to 166 reference PfEMP1 fragments during a dry and subsequent malaria transmission season in Malian adults. Malian adult sera had PfEMP1 serologic responses throughout the year, with decreased reactivity to a small subset of PfEMP1 fragments during the dry season and increases in reactivity to a different subset of PfEMP1 fragments during the subsequent peak malaria transmission season, especially for intracellular PfEMP1 domains. For some individuals, PfEMP1 serologic responses increased after the dry season, suggesting antigenic switching during asymptomatic infection. Adults were more likely to experience variable serorecognition of CD36-binding PfEMP1s than non-CD36-binding PfEMP1s that bind EPCR or ICAM-1, which remained serorecognized throughout the year. Sustained seroreactivity to non-CD36-binding PfEMP1s throughout adulthood amid seasonal fluctuation patterns may reflect underlying protective severe malaria immunity and merits further investigation.

## Introduction

In regions with high malaria transmission, children under five years of age are most vulnerable to severe *Plasmodium falciparum* disease^1^. Older children in these areas acquire immunity to clinical malaria^1^, leading to predominantly asymptomatic malaria infections in adulthood. Although non-sterile natural immunity to malaria is incompletely understood, acquiring antibodies to variant surface antigens (VSA)—proteins expressed on the infected red blood cell surface—plays an important role. VSAs mediate microvascular sequestration of infected red blood cells via host receptor binding^2^, permitting immune system evasion and parasite propagation.

*Plasmodium falciparum* erythrocyte membrane protein-1s (PfEMP1s) comprise the major VSA family implicated in severe malaria pathogenesis and are believed to be the main targets of protective immunity against clinical malaria^3–5^. PfEMP1s are encoded by ~ 60 *var* genes per genome, each containing an upstream promoter sequence and two exons that code for a highly variable extracellular human receptor-binding domain and a relatively conserved intracellular cytoplasmic tail or acidic terminal segment (ATS) residing within the erythrocyte. The extracellular region extrudes into the plasma from the erythrocyte surface and is composed of cysteine-rich interdomain regions (CIDRs) and Duffy binding-like (DBL) domains. The DBL*α* domain includes a 300–400 amino acid sequence unique to individual *var* genes that has been used as a fingerprint “tag” in clinical studies^6–9^. PfEMP1s have previously been divided into five subgroups according to chromosomal location, upstream promoter sequence, and direction of transcription: A, B/A, B, B/C, and C^10,11^. *var* genes have extreme sequence diversity and PfEMP1s have different antigenic and adhesion properties enabling binding to a range of host endothelium receptors. The CIDR*α* domains of some PfEMP1s bind CD36, the most common target receptor of PfEMP1s^11–14^. CD36 is a leukocyte differentiation antigen and scavenger receptor involved in fatty acid metabolism, phagocytosis, and angiogenesis^15–17^. PfEMP1s binding CD36 via CIDR*α*2 domains have been associated with uncomplicated or asymptomatic malaria^18,19^, whereas PfEMP1s binding the endothelial protein C receptor (EPCR)^20^ via CIDR*α*1 domains and PfEMP1s binding the intercellular adhesion molecule-1 (ICAM-1) via DBL$\beta$ domains have been associated with severe malaria^21–27^. Mathematical models suggest that immunity to severe malaria is acquired relatively rapidly in early childhood in malaria-endemic regions, while immunity to uncomplicated malaria is acquired much more slowly^28^. Studies in Tanzanian and Malian children have shown that antibodies to domains of EPCR-binding PfEMP1s are acquired prior to antibodies to other PfEMP1 domain types, supporting the notion that parasites expressing EPCR-binding PfEMP1s are linked to severe malaria in naïve individuals^29,30^. Conversely, parasites that express less pathogenic PfEMP1s may dominate future infections^30^.

Protein microarrays are a powerful tool to measure seroreactivity to a large number of PfEMP1s simultaneously^31^. We used a custom protein microarray to analyze seroreactivity changes to a diverse group of PfEMP1 proteins longitudinally in Malian adults. We hypothesized that adults with lifelong exposure to *P. falciparum* acquire serorecognition of most PfEMP1 antigens and maintain significant PfEMP1 antibody responses throughout the year, particularly to non-CD36-binding PfEMP1s, reflecting sustained protection against severe disease. We also predicted that PfEMP1 serologic responses decrease during the dry season and increase during the malaria transmission season due to the intense, seasonal nature of parasite exposure in Mali.

## Methods

As described previously^32^, microarray construction^33,34^ included (1) polymerase chain reaction amplification of complete or partial *P. falciparum* open reading frames, (2) recombination cloning in *Escherichia coli*, (3) in vitro transcription and translation, and (4) chip printing. Each microarray contained standard controls, as described elsewhere^7^. PfEMP1 fragments were selected for inclusion on the microarray based on their successful amplification and cloning.

A representative set of 166 PfEMP1 protein fragments from the *Plasmodium falciparum* reference strain 3D7 were included on the microarray (see Supplemental Dataset)^35^. We have previously published a detailed description of this microarray and the locations of each of these fragments on 3D7 PfEMP1s^35^. This microarray was probed with sera from 18 adults aged 18–52 years old from the control arm of a malaria vaccine trial in Bandiagara, Mali^36^. Participants were predominantly from the Dogon ethnic group and included 14 males and four females. Malaria transmission in Bandiagara is strictly seasonal with no transmission in the dry season (January–May) and up to 60 infected mosquito bites per person per month during peak malaria transmission (June–December)^37,38^ . Sera from the 18 adults were collected at three time points: December 2004, the start of the dry season (pre-dry season); June 2005, the start of the malaria transmission season (pre-malaria transmission season); and December 2005, the end of the malaria transmission season (post-malaria transmission season). Serum samples from 11 North American, malaria-naïve adult blood donors were used as controls.

For analyses, we grouped PfEMP1 fragments based on the two or three domain composition linked to a known human receptor binding target^39^. A PfEMP1 fragment that contained a domain known to bind EPCR or ICAM-1 or a domain that is a component of a domain cassette known to bind EPCR or ICAM-1 was included in the EPCR/ICAM-1-binding subgroup. Similarly, a PfEMP1 fragment that included a domain known to bind CD36 or a domain that is a component of a domain cassette with a CD36-binding member was included in the CD36-binding subgroup. A PfEMP1 fragment containing domains that typically follow the CD36-binding domain cassette was included in the “CD36-Associated” subgroup. PfEMP1 fragments that did not fall into any of these categories were included in the Miscellaneous subgroup. The remaining protein fragments included DBL$\alpha$ tags, VAR2CSA fragments, VAR1 and VAR3 fragments, and ATS-containing fragments.

Serum samples were obtained from consenting adults in compliance with the International Conference on Harmonization Good Clinical Practices, the Declaration of Helsinki, and Malian regulatory requirements (ClinicalTrials.gov NCT00308061)^36^. Clinical trial protocols were approved by institutional review boards of the University of Bamako Faculty of Medicine, the University of Maryland, Baltimore, and the US Army Surgeon General. Written informed consent was obtained for screening and enrollment in the clinical trial. Verbal consent of illiterate subjects was verified by independent witnesses and documented using thumbprints.

Seroreactivity, measured by microarray fluorescence intensity, and serorecognition, defined as a fluorescence intensity greater than or equal to two standard deviations above the arithmetic mean of North American control sera, were determined for each extracellular and intracellular PfEMP1 fragment and for each DBL*α* tag for each serum sample at each time point. DBL*α* tags were treated as separate from extracellular PfEMP1 fragments in statistical analyses. Seroprevalence refers to the proportion of serum samples that recognized an individual PfEMP1 fragment or group of fragments at a given time point^32^. “Highly seroprevalent” refers to PfEMP1 fragments serorecognized by at least 90 percent of adult serum samples. Seroreactivity trends across PfEMP1 fragments are reported as the mean percentage of fragments ± standard deviation.

Comparisons of seroreactivity and serorecognition were made for both fragments and for serum samples between time points. Unmatched comparisons between PfEMP1 seroreactivities of Malian adult sera and North American malaria-naïve controls were carried out by the Kolmogorov–Smirnov Test to determine group serorecognition at a given time point. Paired comparisons of PfEMP1 seroreactivity between time points were carried out by the Wilcoxon matched-pairs signed-rank test. For paired comparisons of the extent of seasonal change in CD36-binding and non-CD36-binding PfEMP1 seroreactivity seen in Fig. 2, the absolute value of the median change for each individual was calculated and compared between subgroups via the Wilcoxon matched-pairs signed-rank test.

Paired comparisons of PfEMP1 serorecognition between time points were carried out by the two-tailed McNemar test or the two-tailed binomial test for low sample size comparisons. Unmatched comparisons of serorecognition between time points were carried out by the ${\chi }^{2}$ test. Comparisons between observed and expected numbers of fragments in PfEMP1 subgroups were also carried out by ${\chi }^{2}$ test or Fisher’s exact test for low sample size comparisons. ${R}^{2}$ values were used to determine correlations between PfEMP1 percent recognition and age. Significance was defined as *P* < 0.05 for all statistical tests, without adjustment for multiple comparisons, consistent with previous microarray analyses^32,35,40–42^.

### Presented in Part

This information has been previously presented at the Student Research Forum in July 2018 in Baltimore, Maryland as part of the University of Maryland School of Medicine Scholar Program, at Bioscience Research Day 2018 in November 2018 in College Park, Maryland as an undergraduate, and at the American Society of Tropical Medicine and Hygiene Annual Meeting in November 2019 in National Arbor, Maryland (abstract number: 361).

## Results

### High levels of PfEMP1 serologic responses across the year

**Figure 1:**
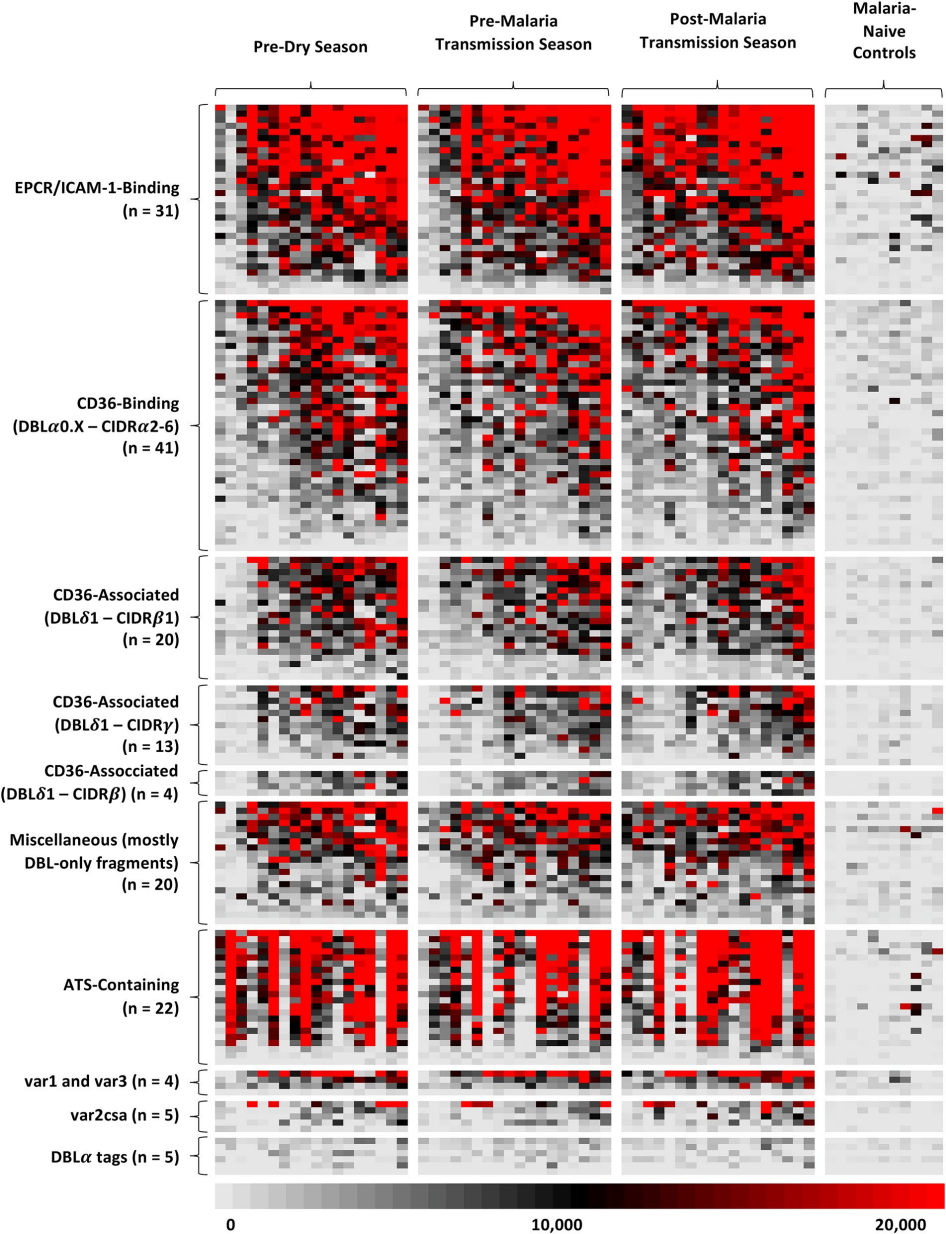
166 *Plasmodium falciparum* erythrocyte membrane protein-1 (PfEMP1) fragments encoded by the 3D7 reference genome were printed on a protein microarray and probed with serum samples from 18 adults aged 18 to 55 from Bandiagara, Mali and 11 North American malaria-naive adult controls. Signal intensities were quantified and represented on the heat map, ranging from weak (*grey*) to intermediate (*black*) to intense (*red*). Columns represent the signal intensities for individual serum samples, and rows represent the signal intensities for individual PfEMP1 fragments. PfEMP1 microarrays were probed with serum samples from malaria-endemic individuals at three time points throughout the year—pre-dry season (left), pre-malaria season (left center), and post-malaria season (right center). Within the control group (right) and for each time point, individual serum samples are sorted by increasing mean fluorescence intensity. PfEMP1 fragments are grouped according to domain composition and host receptor binding association. “CD36-associated” refers to a PfEMP1 fragment downstream from the CIDR*α* domain of a known CD36-Binding 3D7 PfEMP1. Within each group, PfEMP1 variants are ordered by decreasing mean fluorescence intensity. *CIDR* cysteine-rich interdomain regions, *DBL* Duffy binding-like, *DBLα tag* Duffy binding-like alpha tag, *ATS* acidic terminal segment.

A microarray featuring 138 extracellular and 22 intracellular 3D7 PfEMP1 fragments and six 3D7 DBL*α* tags, was probed with Malian sera from 14 males and four females aged 18–52 years. No participants were hospitalized for severe malaria during the study. We detected a range of antibody responses to all fragments, including intracellular and extracellular PfEMP1 fragments (Fig. 1). As a group, Malian adults maintained broad serorecognition of PfEMP1s across the year, including the vast majority of extracellular and intracellular PfEMP1 fragments pre-dry season (92.8% and 86.4%, respectively), pre-malaria transmission season (94.2% and 95.5%, respectively), and post-malaria transmission season (95.7% and 95.5%, respectively). Most individuals possessed broad serorecognition of extracellular and intracellular PfEMP1 fragments at all three time points (pre-dry season: 72.2% ± 21.4% and 72.5% ± 23.6% of PfEMP1 fragments; pre-malaria transmission season: 74.0% ± 23.0% and 64.9% ± 26.6%; post-malaria transmission season: 79.1% ± 13.2% and 75.3% ± 26.7%, respectively) (Fig. S1). Notably, several individuals consistently lacked serorecognition of intracellular PfEMP1s, despite broad serorecognition of extracellular PfEMP1s (Fig. 1). The extent of serorecognition of PfEMP1s, including subsets, was not correlated with age at any time point (Table S1).

### Group serologic responses

Over the course of both the dry season and malaria transmission season, Malian adults, as a group, maintained serorecognition of the vast majority of PfEMP1s, but more frequently had fluctuations in PfEMP1 seroreactivity (Table 1). Adults had decreased seroreactivity to 14 PfEMP1 fragments in the dry season and increased seroreactivity to 8 PfEMP1 fragments in the malaria transmission season, with a significant decline in the group’s seroreactivity to intracellular PfEMP1 fragments in the dry season and an increase in the malaria transmission season (Table 1). The group experienced a trend towards decline in seroreactivity to extracellular PfEMP1 fragments in the dry season but maintained seroreactivity levels within the malaria transmission season (Table 1).

**Table 1:** Changes in serorecognition and seroreactivity for Malian adults as a group to 138 extracellular and 22 intracellular *Plasmodium falciparum* erythrocyte membrane protein-1 (PfEMP1) fragments across both seasons and the entire year.

Serorecognition	Maintained serorecognition	Stayed unrecognized	Gained serorecognition	Lost serorecognition	*P* value
**Dry Season**					
Extracellular	92.0% (n = 127)	5.1% (n = 7)	2.2% (n = 3)	0.7% (n = 1)	0.313
Intracellular	86.4% (n = 19)	4.5% (n = 1)	9.1% (n = 2)	0% (n = 0)	0.250
DBLα tags	33.3% (n = 2)	66.7% (n = 4)	0% (n = 0)	0% (n = 0)	
**Malaria Transmission Season**					
Extracellular	94.2% (n = 130)	4.3% (n = 6)	1.4% (n = 2)	0.7% (n = 0)	0.250
Intracellular	95.5% (n = 21)	4.5% (n = 1)	0% (n = 0)	0% (n = 0)	1.000
DBLα tags	16.7% (n = 1)	66.7% (n = 4)	0% (n = 0)	16.7% (n = 1)	
**Entire Year**					
Extracellular	92.8% (n = 128)	4.3% (n = 6)	2.9% (n = 4)	0% (n = 0)	0.063
Intracellular	86.4% (n = 19)	4.5% (n = 1)	9.1% (n = 2)	0% (n = 0)	0.250
DBLα tags	16.7% (n = 1)	66.7% (n = 4)	0% (n = 0)	16.7% (n = 1)	

Seroreactivity	Maintained		Increased	Decreased	*P* value
**Dry Season**					
Extracellular	93.5% (n = 129)		1.4% (n = 2)	5.1% (n = 7)	0.090
Intracellular	72.7% (n = 16)		0% (n = 0)	27.3% (n = 6)	0.016
DBLα tags	83.3% (n = 5)		0% (n = 0)	16.7% (n = 1)	
**Malaria Transmission Season**					
Extracellular	97.8% (n = 135)		2.2% (n = 3)	0% (n = 0)	0.125
Intracellular	77.3% (n = 17)		22.7% (n = 5)	0% (n = 0)	0.031
DBLα tags	83.3% (n = 5)		0% (n = 0)	16.7% (n = 1)	
**Entire Year**					
Extracellular	95.7% (n = 132)		2.9% (n = 4)	1.4% (n = 2)	0.344
Intracellular	95.5% (n = 21)		4.5% (n = 1)	0% (n = 1)	0.500
DBLα tags	66.7% (n = 4)		0% (n = 0)	33.3% (n = 2)	

*P* values reflect whether changes across the given time interval are significant and are given by the binomial test; Wilcoxon matched-pairs signed-rank test determined serorecognition status across time periods for PfEMP1 fragments.

Across the year as a whole—spanning the beginning of the dry season to the end of the malaria transmission season—adults as a group maintained serorecognition of the vast majority of PfEMP1s, with a trend toward increased serorecognition counts of extracellular PfEMP1 fragments that approached significance (Table 1). In contrast to findings in each of the dry and malaria transmission seasons, adults experienced no change in seroreactivity levels to the vast majority of both intracellular PfEMP1 fragments and extracellular PfEMP1 fragments (95.5% and 95.7%, respectively) (Table 1). The group had increased seroreactivity to a minority of extracellular and intracellular PfEMP1 fragments (4 and 1, respectively). The group had decreased seroreactivity to a comparable number of extracellular and intracellular PfEMP1 fragments (2 and 0, respectively) (Table 1).

### Individual serologic responses

**Figure 2:**
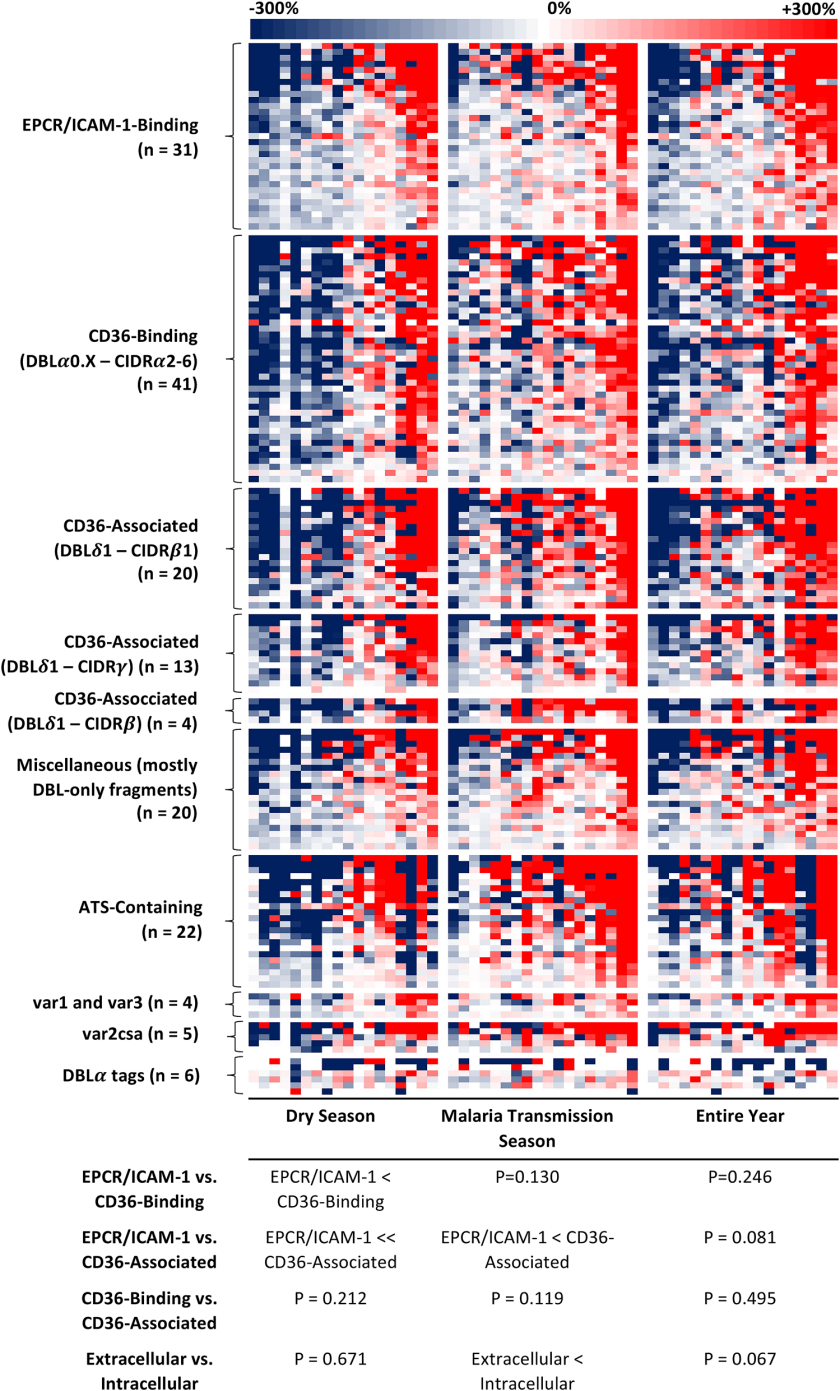
Sera from Malian adults had changes in *Plasmodium falciparum* erythrocyte membrane protein-1 (PfEMP1) seroreactivity across the dry season, malaria transmission season, and entire year. The heat map indicates fluorescence intensity for each of 166 PfEMP1 fragments adjusted by the change in proportion to the serorecognition threshold for that fragment (defined as two standard deviations above the arithmetic mean of North American control sera) for each Malian adult over the course of the dry season (left), malaria transmission season (center), and the entire year (right). PfEMP1 fragments (represented by rows) are grouped according to domain composition and host receptor binding association. PfEMP1 fragments within each subgroup are ordered by decreasing cumulative serologic responses (weighed by the count of changes > 300%), while individuals are ordered by increasing serologic responses (weighed by the count of changes > 300%). Red indicates an *increase* in serologic response, blue indicates a *decrease* in serologic response, and white indicates *no change* in serologic response over the course of the respective season or entire year. When the serorecognition threshold was zero, a one was substituted to calculate the serorecognition threshold proportion. The table below the heat map summarizes paired comparisons of the extent of seroreactivity change between CD36-binding and associated PfEMP1 fragments and endothelial protein C receptor- (EPCR) and intercellular adhesion molecule-1 (ICAM-1)-binding PfEMP1 fragments. “CD36-associated” refers to a PfEMP1 fragment downstream from the CIDR*α* domain of a known CD36-Binding 3D7 PfEMP1. ‘>>>’ and ‘<<<’ indicate *P* < 0.001, ‘>>’ and ‘<<’ indicate *P* < 0.01, and ‘>’ and ‘<’ indicate *P* < 0.05 in the Wilcoxon matched-pairs signed-rank test. *CIDR* cysteine-rich interdomain regions, *DBL* Duffy binding-like, *DBLα tag* Duffy binding-like alpha tag.

Changes in PfEMP1 seroreactivity across the dry season were not consistent across all adults but tended to go in the same direction for most PfEMP1s for a given individual (74.9% $\pm$ 14.2% of PfEMP1s changed in the same direction per adult; Fig. 2), which for the majority of adults was a decrease in seroreactivity [10 of 18 (55.6%) adults]. Likewise, changes in PfEMP1 seroreactivity across the malaria transmission season varied between adults but tended to go in the same direction for most PfEMP1s for a given individual (70.7% ± 15.2% of PfEMP1s changed in the same direction per adult; Fig. 2), which for the majority of adults was an increase in seroreactivity [10 of 18 (55.6%) adults]. These seasonal changes were more pronounced for both CD36-binding PfEMP1 fragments and CD36-associated PfEMP1 fragments compared to EPCR/ICAM-1-binding PfEMP1s (Fig. 2). Seasonal changes were also more pronounced for intracellular compared to extracellular PfEMP1 fragments (Fig. 2).

**Figure 3:**
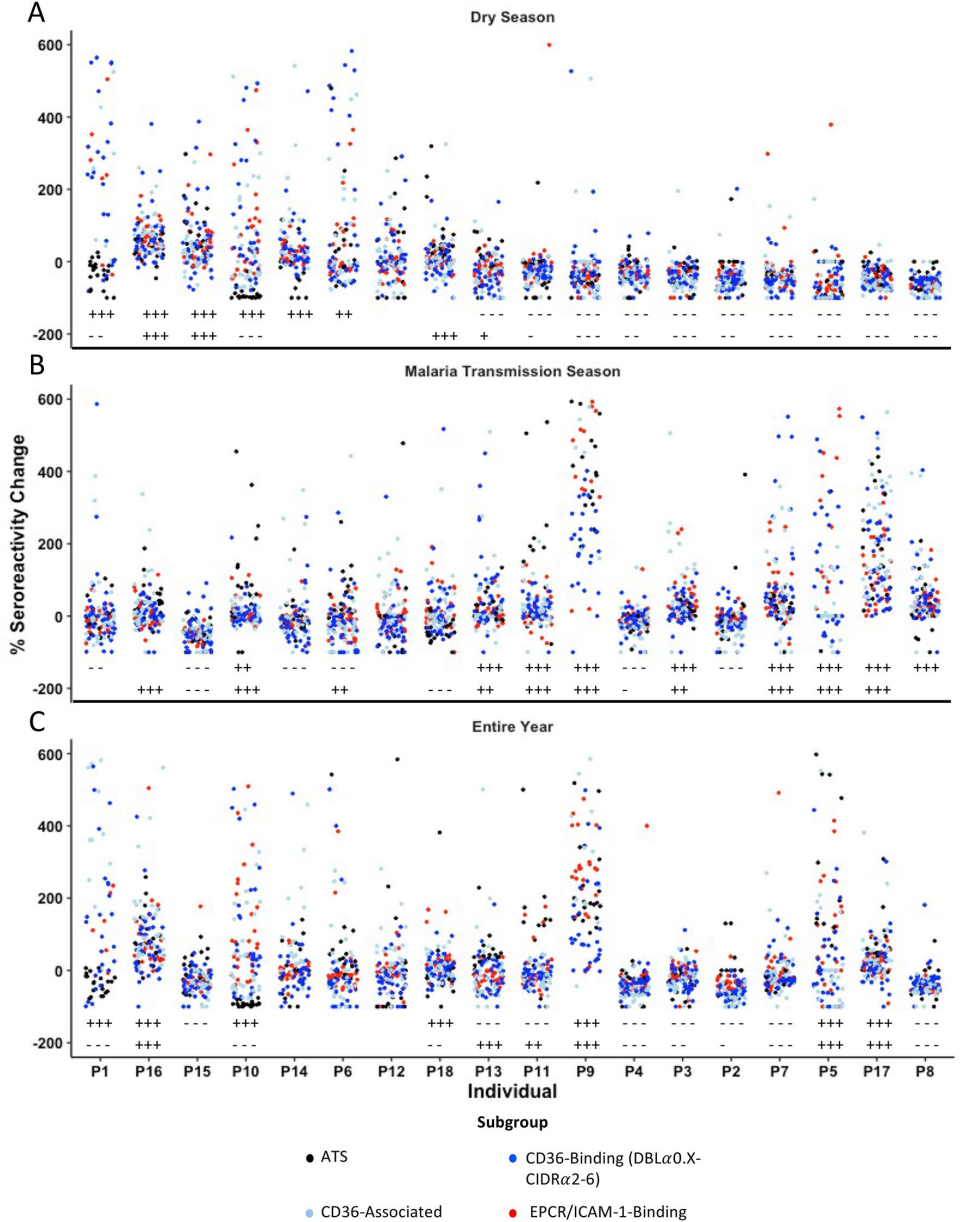
Percent changes in fluorescence intensity for each of the 166 *Plasmodium falciparum* erythrocyte membrane protein-1 (PfEMP1) fragments for each adult over the course of the dry season (**A**), malaria transmission season (**B**), and entire year (**C**). PfEMP1 fragments are separated into intracellular PfEMP1s (black) PfEMP1s (red), CD36-associated PfEMP1s (light blue), CD36-binding PfEMP1s (blue), and EPCR/ICAM-1-binding PfEMP1s. Individuals in all three panels are organized by decreasing median percent change in fluorescence intensity across the dry season. ‘+++’ and ‘−−−’ indicate *P* < 0.001, ‘++’ and ‘− −’ indicate *P* < 0.01, and ‘+’ and ‘−’ indicate *P* < 0.05 in the Wilcoxon matched-pairs signed-rank test for extracellular PfEMP1s (top row) and intracellular PfEMP1s (bottom row). Values above 600% flourescence intensity change are not shown, resulting in 89.2% of all data being displayed. “CD36-associated” refers to a PfEMP1 fragment downstream from the CIDR*α* domain of a known CD36-Binding 3D7 PfEMP1. *CIDR* cysteine-rich interdomain regions, *DBL* Duffy binding-like, *ATS* acidic terminal segment, *EPCR* Endothelial protein C receptor, *ICAM-1* Intercellular adhesion molecule-1.

Individual adults maintained similar serologic responses to most PfEMP1 fragments across the dry season and malaria transmission season but experienced some decreased responses in the dry season (seroreactivity: Fig. 3A; serorecognition: Fig. S1A) and increased responses in the malaria transmission season (seroreactivity: Fig. 3B; serorecognition: Fig. S1B). The majority of adults maintained serorecognition of most extracellular PfEMP1 fragments and intracellular PfEMP1 fragments across the dry season and across the malaria transmission season. More adults had decreases in seroreactivity to extracellular (n = 10 adults) or intracellular PfEMP1 fragments (n = 11 adults) in the dry season compared to the malaria transmission season (n = 6 and 3 adults, respectively). In contrast, a higher number of adults had increases in seroreactivity to extracellular (n = 9 adults) or intracellular PfEMP1 fragments (n = 10 adults) in the malaria transmission season (Fig. 3B) compared to the dry season (n = 6 and 4 adults, respectively) (Fig. 3A). Serorecognition of extracellular and intracellular PfEMP1 fragments mirrored this trend (Fig. S1). Notably, adults had more maintained seroreactivity to extracellular (n = 3 adults) or intracellular PfEMP1 fragments (n = 5 adults) in the malaria transmission season (Fig. 3B) compared to the dry season (n = 2 and 3 adults, respectively) (Fig. 3A). The same was true of extracellular PfEMP1 serorecognition (Fig. S1). Seasonal fluctuations in the serorecognition of PfEMP1 fragments was more common for extracellular fragments than intracellular fragments (Fig. S1).

Across the entire year, most adults maintained serorecognition of most extracellular (n = 14 adults) and intracellular PfEMP1 fragments (n = 12 adults). About a third of adults experienced overall increases in seroreactivity to extracellular or intracellular PfEMP1 fragments (n = 7 and 6) (Fig. 3C). A similar proportion of adults experienced overall decreases in seroreactivity to extracellular or intracellular PfEMP1 fragments (n = 8 and 8) (Fig. 3C). This trend was also observed for serorecognition of extracellular and intracellular PfEMP1 fragments (Fig. S1C).

### Oscillations in serologic responses

**Figure 4:**
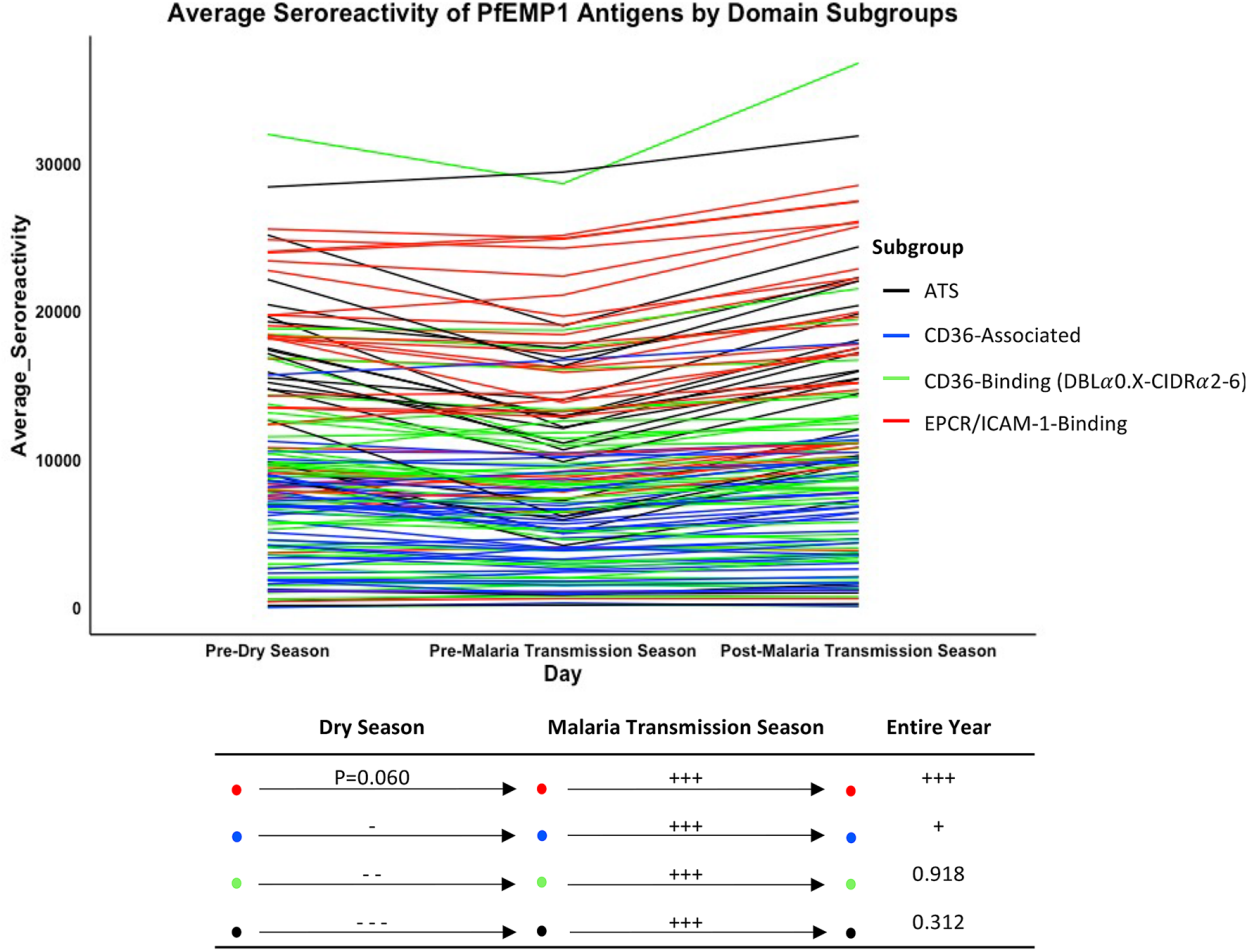
Mean seroreactivity trajectories for individual PfEMP1 fragments across three time points across the year. Individual lines are colored by PfEMP1 subgroup and represent adult mean seroreactivity to the PfEMP1 fragment at three time points: pre-dry season, pre-malaria transmission season, and post-malaria transmission season. The diagram below depicts changes in mean seroreactivity to four subgroups across the dry season (left), malaria transmission season (center), and entire year (right). ‘+++’ and ‘−−−’ indicate *P* < 0.001, ‘++’ and ‘− −’ indicate *P* < 0.01, and ‘+’ and ‘−’ indicate *P* < 0.05 in the Wilcoxon matched-pairs signed-rank test. “CD36-associated” refers to a PfEMP1 fragment downstream from the CIDR*α* domain of a known CD36-Binding 3D7 PfEMP1. CIDR: cysteine-rich interdomain regions; DBL: Duffy binding-like; ATS: acidic terminal segment; EPCR: Endothelial protein C receptor; ICAM-1: Intercellular adhesion molecule-1.

Declines in seroreactivity and serorecognition in the dry season were frequently met with increases in the following malaria transmission season. Adults as a group had decreased seroreactivity to 14 PfEMP1s (including six intracellular, seven extracellular PfEMP1 fragments, and one DBL*α* tag) in the dry season. In the following malaria transmission season, adults had increased seroreactivity to three of these PfEMP1 fragments and maintained seroreactivity to an additional 11 fragments. The group’s average seroreactivity to intracellular PfEMP1 fragments and CD36-binding and associated PfEMP1 fragments declined in the dry season and increased in the malaria transmission season but remained at similar levels across the entire year (Fig. 4). The group’s average seroreactivity to EPCR/ICAM-1-Binding PfEMP1 fragments trended toward a decline in the dry season (Wilcoxon matched pairs signed-ranks test; *P* = 0.060) and increased in the malaria transmission season, resulting in a significant increase across the year as a whole (Fig. 4).

On an individual level, almost all adults with decreases in serologic responses to extracellular PfEMP1s in the dry season (seroreactivity: Fig. 3A and serorecognition: Fig. S1A) maintained or increased responses in the following malaria transmission season (seroreactivity: Fig. 3B and serorecognition: Fig. S1B). Similarly, adults that had increases in serologic responses to extracellular PfEMP1s in the dry season (Figs. 3A and S1A) had either declines or maintained PfEMP1 responses in the malaria transmission season (Figs. 3B and S1B). Seasonal serologic responses to intracellular PfEMP1 fragments also mirrored these trends (Figs. 3 and S1).

### Maintained serologic responses to group A and non-CD36-binding PfEMP1s across seasons

We identified subsets of PfEMP1 fragments that remained highly seroprevalent across the dry season (six extracellular and two intracellular PfEMP1 fragments), malaria transmission season (16 extracellular and one intracellular PfEMP1 fragments), and entire year (18 extracellular and two intracellular PfEMP1 fragments) (see Supplemental Dataset 1). Within these seroprevalent PfEMP1 subsets, the observed numbers of EPCR/ICAM-1-binding PfEMP1 fragments were larger than expected by chance in the dry season, malaria transmission season and entire year, though these differences were not statistically significant (Fig. S2). Additionally, within these seroprevalent subsets, all Malian adults had serorecognition of particular PfEMP1 fragments across the dry season, malaria transmission season, and entire year (Table 2).

**Table 2:** Subsets of PfEMP1 fragments serorecognized by all Malian adults across the dry and malaria transmission seasons.

Season	Fragment	3D7 Protein	Subgroup	Extracellular or Intracellular
Dry season	PF08_0107_ATS	PF08_0107	ATSB	Intracellular
Malaria transmission season	PF08_0107_ATS PF08_0141_3 PF11_0008e1s1 PF11_0521_3	PF08_0107 PF08_0141 PF11_0008 PF11_0521	ATSB Miscellaneous EPCR/ICAM-1-binding EPCR/ICAM-1-binding	Intracellular Extracellular Extracellular Extracellular
Entire year	PF08_0107_ATS PF08_0103_1 MAL6P1252_1	PF08_0107 PF08_0103 MAL6P1252	ATSB CD36-Binding (DBLa0.X-CIDR*α*2–6) CD36-Binding (DBLa0.X-CIDR*α*2–6)	Intracellular Extracellular Extracellular

**Figure 5:**
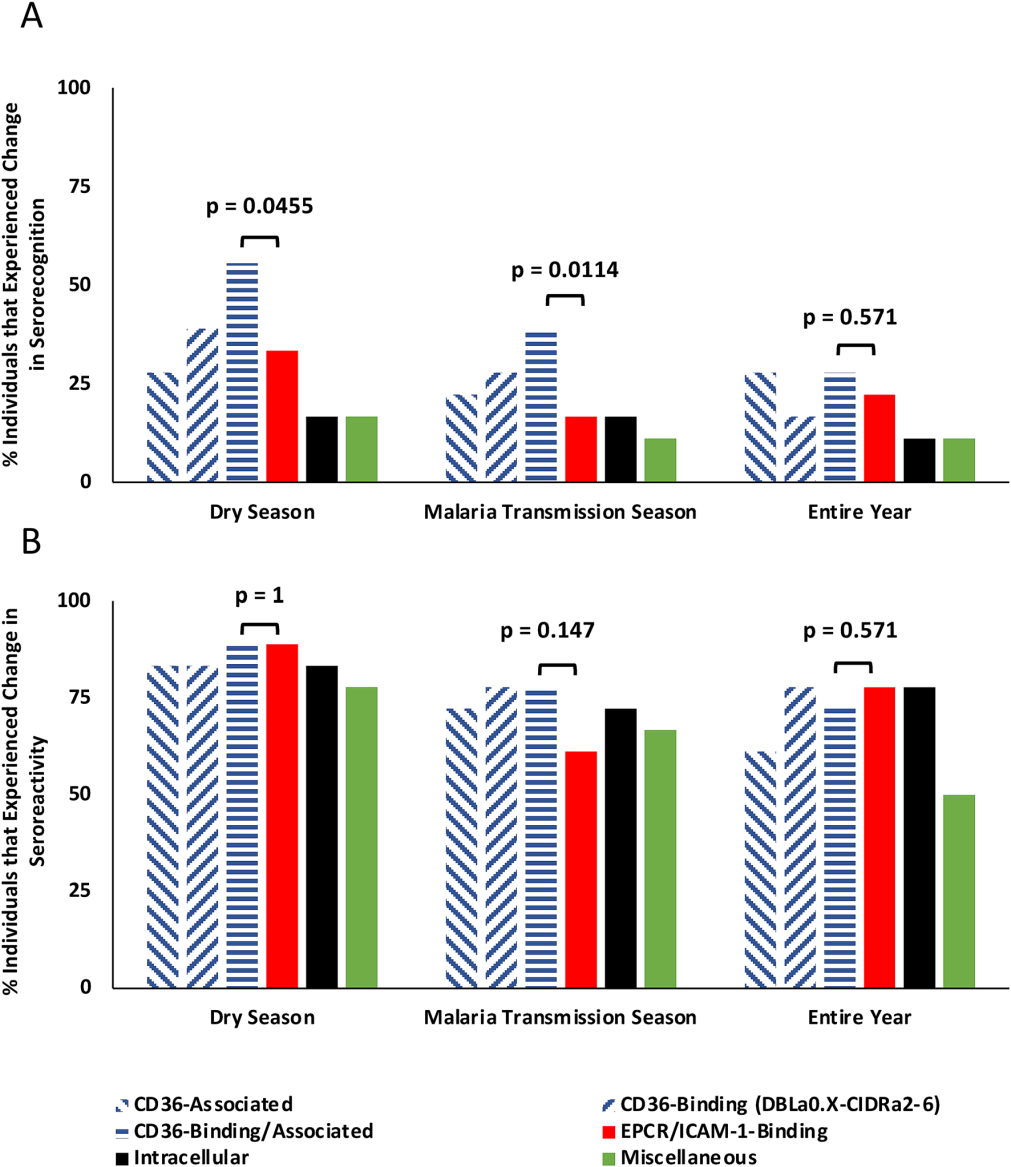
Serorecognition of the combined subgroup of CD36-binding and CD36-associated *Plasmodium falciparum* erythrocyte membrane protein-1 (PfEMP1) fragments changed more frequently than did serorecognition of EPCR/ICAM-1-binding PfEMP1s. Depicted are the proportions of individuals that experienced change in serorecognition (**A**) and seroreactivity (**B**) of the combined subgroup of CD36-binding and CD36-associated (horizontal blue stripes) and EPCR/ICAM-1-binding PfEMP1s (red) PfEMP1s (determined by the McNemar’s or binomial test) across the dry season, malaria transmission season (MTS), and entire year. CD36-binding, CD36-associated, and intracellular subgroups are shown separately for comparison. *P* values indicate ${\chi }^{2}$ comparisons between the combined CD36-binding/associated subgroup (actual) and EPCR/ICAM-1-binding PfEMP1s counts (expected). “CD36-associated” refers to a PfEMP1 fragment downstream from the CIDR*α* domain of a known CD36-Binding 3D7 PfEMP1. *CIDR* cysteine-rich interdomain regions, *DBL* Duffy binding-like, *ATS* acidic terminal segment, *EPCR* Endothelial protein C receptor, *ICAM-1* Intercellular adhesion molecule-1.

In individuals, patterns of serorecognition for the combined subgroup of CD36-binding and CD36-associated PfEMP1 fragments changed more frequently across each season than did serorecognition for EPCR/ICAM-1-binding PfEMP1 fragments, although this was not significant when the entire year was considered (Fig. 5A). Seroreactivity differences for the combined subgroup of CD36-binding and CD36-associated PfEMP1 fragments versus EPCR/ICAM-1-binding extracellular PfEMP1 fragments across each season were not significant (Fig. 5B).

## Discussion

We determined antibody responses to 166 3D7 PfEMP1 fragments for 18 adults residing in a region of intense seasonal malaria transmission at three time points over the course of a year. Adults maintained broad serorecognition of PfEMP1s across the dry season and the following malaria transmission season. We noted decreased adult PfEMP1 serologic responses in the dry season and subsequent maintained or increased serologic responses in the malaria transmission season, likely reflecting increased exposure to the malaria parasite and their PfEMP1s in the malaria transmission season.

These findings suggest that parasite exposure may be important for both sustained and increased serologic responses to PfEMP1s. Interestingly, some adults developed increased seroreactivity to and serorecognition of PfEMP1s across the dry season, when they presumably have almost no exposure to infected mosquitoes. These increased PfEMP1 serologic responses may be due to continued carriage of blood stage malaria parasites throughout the dry season and suggest antigenic switching in the parasite (we did not monitor adults in the study for asymptomatic parasite carriage). Indeed, a study that compared asymptomatic Malian children in the dry season to symptomatic children in the malaria transmission season found distinct *P. falciparum* gene expression between the two seasons^43^. Adults in areas of seasonal malaria transmission such as Ghana^44^, Senegal^45^, and Mali^46^ have been shown to harbor asymptomatic malaria parasites in the dry season.

We also noted seasonal oscillation patterns in PfEMP1 seroreactivity and serorecognition. Individuals that experienced decreased or increased PfEMP1 serologic responses in the dry season tended to have changes in the opposite direction in the malaria transmission season. These serologic fluctuations occurred for extracellular PfEMP1 fragment subgroups and intracellular PfEMP1 fragments. While the group’s average seroreactivity to the extracellular CD36-binding and CD36-associated PfEMP1 subgroups remained constant across the year overall after decreasing in the dry season and increasing in the malaria transmission season, the group experienced increased seroreactivity to the EPCR/ICAM-1-binding PfEMP1s across the entire year. Additionally, seroreactivity changes were more pronounced for CD36-binding and CD36-associated PfEMP1 fragments across the dry and malaria transmission seasons than they were for EPCR/ICAM-1-binding PfEMP1s, but this was not the case across the entire year. We also noted pronounced seasonal seroreactivity changes to intracellular PfEMP1 fragments, which are encoded by a relatively conserved exon in *var* genes. It may not be surprising that adults as a group had shared serologic responses to intracellular PfEMP1 fragments, which they are more likely to have been exposed to previously, given sequence conservation. In contrast, adults as a group had less consistent responses to extracellular PfEMP1 fragments, which are more diverse and thus less likely to be elicit consistent serologic responses from adults. Likewise, individuals had seasonal fluctuations in serorecognition of these diverse extracellular PfEMP1 fragments.

Individuals maintained serorecognition of EPCR/ICAM-1-binding PfEMP1 fragments more often than they did of the CD36-binding and CD36-associated PfEMP1 fragments. Among highly seroprevalent PfEMP1 fragments, EPCR/ICAM-1-binding PfEMP1s were overrepresented. A previous study of pediatric cerebral malaria cases found increased transcript abundance of PfEMP1 domain cassette eight (DC8) and group A EPCR-binding domains in children with brain swelling, a main cause of mortality in pediatric cerebral malaria^47^. Sustained seroreactivity to EPCR/ICAM-1-binding PfEMP1 fragments included on our microarray, which included domain cassette 8 PfEMP1 fragments, into adulthood may reflect sustained protective clinical immunity to severe malaria. PfEMP1s that bind EPCR and ICAM-1 may lead to the development of severe malaria^26,27,35,48^.

These results suggest a potential model for explaining sustained protection against severe malaria in adults in highly seasonal malaria settings. We found that most adults maintained serorecognition of the majority of PfEMP1s, especially EPCR/ICAM-1-binding PfEMP1s, throughout the study period. This occurred amid some seasonal serologic response fluctuations, during which decreased antibody responses in the dry season were met with maintained or increased antibody responses in the malaria transmission season. Despite not having severe malaria during the study period, these adults maintained serologic responses to PfEMP1s associated with severe disease. Blood stage parasite exposure most likely sustains PfEMP1 serologic responses, including in the dry season, when adults may have asymptomatic parasite carriage. We hypothesize that any malaria exposure—even with asymptomatic infection—sustains serologic responses to non-CD36-binding PfEMP1s in adults. Declines in PfEMP1 serologic responses likely reflect a lack of parasite exposure and could signal increased vulnerability to infection.

A previous study of antibody responses to multiple immunogenic *P. falciparum* proteins (including 19 PfEMP1 fragments) showed only minor increases in serorecognition for adults across a malaria transmission season in Mali^31^. We previously found that for 21 PfEMP1 fragments from eight PfEMP1s, the proportions of adult sera that recognized intracellular and extracellular PfEMP1 fragments across the malaria transmission season did not change^32^. Another study with microarrays featuring 123 recombinant PfEMP1-DBL-α “tags” (a ~ unique 100 amino acid PfEMP1 extracellular region) found similar antibody responses between infected and uninfected adults in Papua New Guinea^7^, a region with limited PfEMP1 genetic diversity^9^. Interestingly, in this Papua New Guinea population, antibody responses against group A PfEMP1s appear to reduce the risk of severe disease in children, supporting the idea that sustained responses to these PfEMP1s may protect adults from severe disease as well^49^. The fluctuation in serologic responses across seasons and between adults observed in the present study demonstrates the importance of including a diverse and broadly representative set of large PfEMP1 fragments when analyzing changes in serologic responses across time periods and between infected and uninfected adults.

Additionally, several adults consistently lacked serorecognition of intracellular PfEMP1 fragments despite high levels of seroreactivity to extracellular PfEMP1 fragments. Seroreactivity to intracellular domains has previously been regarded as a measure of malaria exposure and has been observed to be higher in adults versus children in Mali and Kenya and higher versus extracellular PfEMP1 domains^32,50^. This higher seroreactivity to intracellular domains reflects increased sequence conservation of intracellular PfEMP1 fragments versus extracellular PfEMP1 fragments. Thus, while some extracellular antigens tend to be more seroreactive than intracellular antigens^51^, conserved intracellular portions of a PfEMP1 have been more seroreactive than diverse extracellular portions of the same PfEMP1^32^. These unusually low serologic responses to intracellular PfEMP1 fragments that we have observed in a minority of Malian adults suggests an altered immune response due to an unknown host factor that was not ascertained in this study.

This study had several limitations. The vaccine trial that was the source of sera for this study did not include systematic monitoring for uncomplicated or asymptomatic malaria episodes^36^, so we do not know whether any of the 18 adults experienced such malaria episodes. Our limited sample size of 18 adults also limited our ability to detect subtle trends in serologic responses. Additionally, 20 percent of PfEMP1 domains in the reference strain 3D7 were not successfully expressed and so were not represented on the microarray. Another potential limitation is that there may be seasonality to the predominant PfEMP1s in a community. If this is the case, then seroepidemiological trends may reflect this seasonality, but such PfEMP1 seasonality has not yet been demonstrated.

Future analysis should study PfEMP1 serologic responses over longer time periods and in a large, closely followed cohort of adults in malaria-endemic regions to identify specific PfEMP1 domains that are crucial for conferring protection against clinical malaria.

## Supplementary information

Supplementary Dataset 1.

Supplementary Dataset 2.

Supplementary Information

## Data Availability

The microarray dataset generated for this analysis is included as Supplementary Dataset 2.
