# Supplementary material for: Malian adults maintain serologic responses to virulent PfEMP1s amid seasonal patterns of fluctuation
Source: Sci Rep. 2021 Jul 13;11:14401. doi: 10.1038/s41598-021-92974-7 (PMC8277812; doi:10.1038/s41598-021-92974-7)
Supplement: Supplementary file 3 — Supplementary Information [file 41598_2021_92974_MOESM3_ESM.pdf]

Malian Adults Maintain Serologic Responses to Virulent PfEMP1s Amid Seasonal Patterns of Fluctuation

Noah T. Ventimiglia[1], Emily M. Stucke[1], Drissa Coulibaly[2], Andrea A. Berry[1], Kirsten E. Lyke[1], Matthew B. Laurens[1], Jason A. Bailey[1], Matthew Adams[1], Amadou Niangaly[2], Abdoulaye K. Kone[2], Shannon Takala-Harrison[1], Bourema Kouriba[2], Ogobara K. Doumbo[2†], Phillip L. Felgner[3], Christopher V. Plowe[1], Mahamadou A. Thera[2], Mark A. Travassos[1*]

[1]University of Maryland School of Medicine, Baltimore, MD, United States, [2]University of Sciences, Techniques and Technologies, Bamako, Mali, [3]University of California, Irvine, CA, United States

[†]Deceased

*Corresponding Author: mtravass@som.umaryland.edu

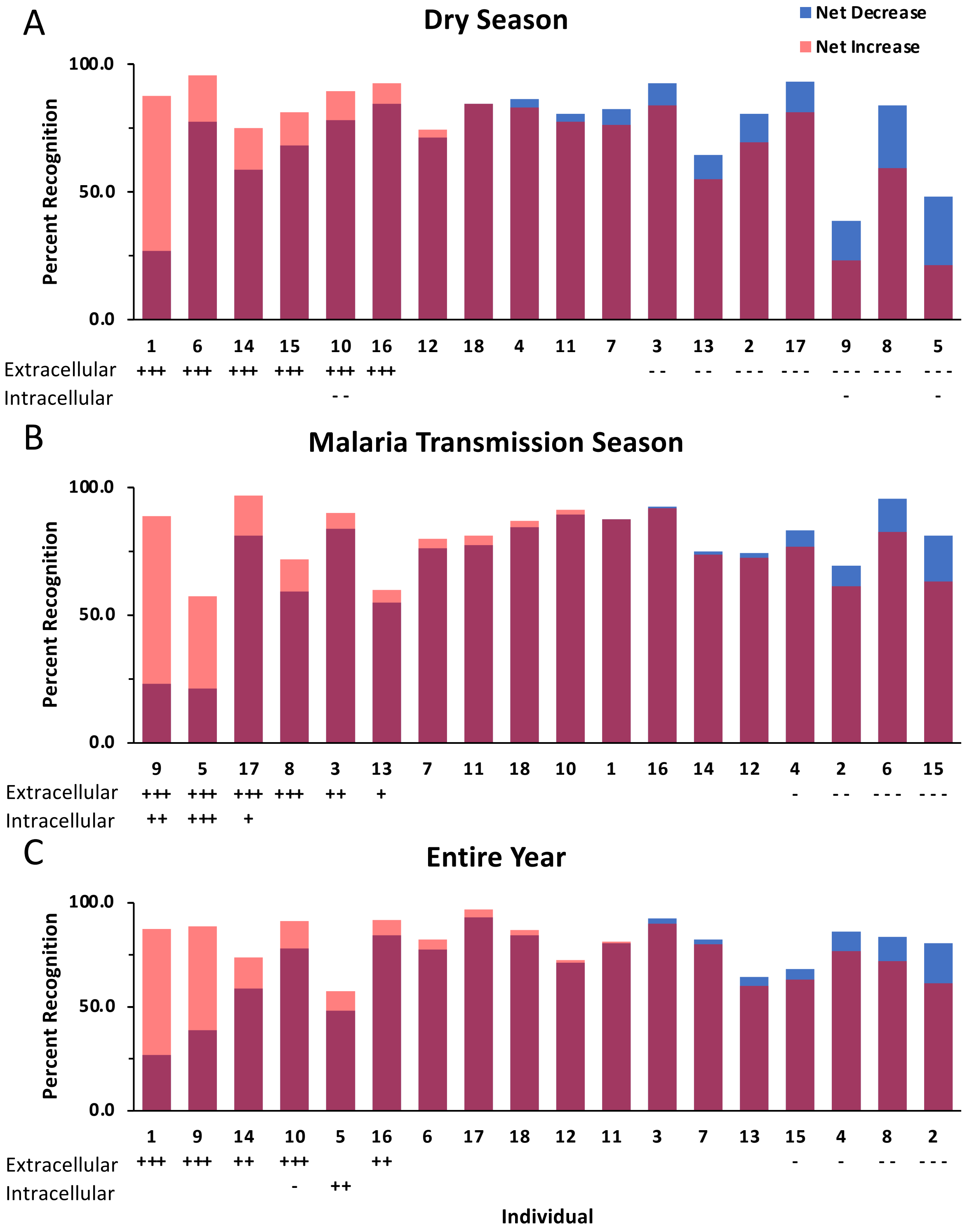

A
Dry Season
Net Decrease
Net Increase
100.0
50.0
0.0
Percent Recognition
1 6 14 15 10 16 12 18 4 11 7 3 13 2 17 9 8 5
Extracellular +++ +++ +++ +++ +++ +++ -
Intracellular - - - -
B
Malaria Transmission Season
9 5 17 8 3 13 7 11 18 10 1 16 14 12 4 2 6 15
Extracellular +++ +++ +++ +++ ++ + - - - - - - - - -
Intracellular ++ +++ +
C
Entire Year
1 9 14 10 5 16 6 17 18 12 11 3 7 13 15 4 8 2
Extracellular +++ +++ ++ +++ ++ - - - - - - -
Intracellular - ++
Individual

Figure S1. Sera from Malian adults had shifts in serorecognition across the dry and malaria transmission seasons. Depicted are the percentages of *Plasmodium falciparum* erythrocyte membrane protein-1 (PfEMP1) fragments that were recognized by each malaria-endemic individual's sera pre- (blue) and post- (orange) dry season (A), malaria transmission season (B), and entire year (C). Serum samples are arranged by decreasing difference in overall PfEMP1 percent recognition. '+++' and '- - -' indicate P < 0.001, '++' and '- -' indicate P < 0.01, and '+' and '-' indicate P < 0.05 by the two-tailed McNemar's test or two-tailed Binomial test for extracellular PfEMP1s (above) and intracellular PfEMP1s (below).

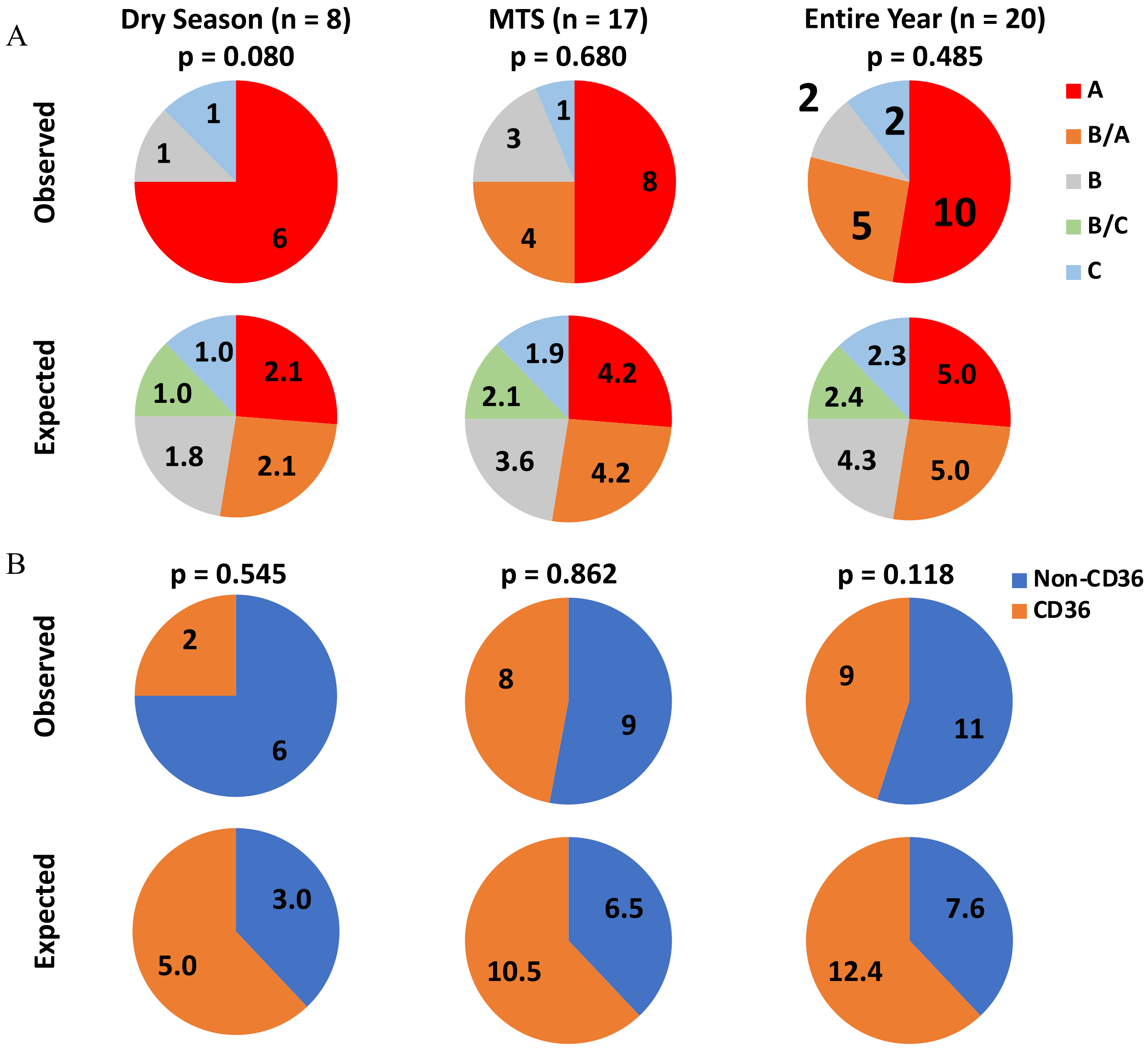
A
Dry Season (n = 8)
p = 0.080
MTS (n = 17)
p = 0.680
Entire Year (n = 20)
p = 0.485
Observed
1
1
6
1
3
8
4
2
2
5
10
A
B/A
B
B/C
C
Expected
1.0
1.0
2.1
1.8
2.1
1.9
2.1
4.2
3.6
4.2
2.3
2.4
5.0
4.3
5.0
B
p = 0.545
p = 0.862
p = 0.118
Non-CD36
CD36
Observed
2
6
8
9
9
11
Expected
3.0
5.0
6.5
10.5
7.6
12.4

Figure S2. Observed numbers of highly seroprevalent Group A (A) and non-CD36-binding (B) *Plasmodium falciparum* erythrocyte membrane protein-1 (PfEMP1) fragments exceeded expected numbers for the dry season (left column), malaria transmission season (MTS) (center column) and entire year (right column). This figure represents the observed numbers (above) and expected numbers (below) of PfEMP1 fragments belonging to different PfEMP1 subgroups among PfEMP1s with high seroprevalence (recognized by $\geq$ 17 individuals). P values are given by the χ^2 test or Fisher's exact test.

Table S1: R^2 values for the correlation of age with proportion of serorecognized PfEMP1 fragments.

	Correlation Coefficient (R^2)		
	**Pre-Dry Season**	**Pre-Malaria Transmission Season**	**Post-Malaria Transmission Season**
**Extracellular (n = 138)**	0.17	-0.32	0.15
EPCR/ICAM-1 (n = 31)	0.21	-0.29	0.07
CD36-Binding (DBLa0.X-CIDRa2-6) (n = 41)	0.18	-0.18	0.37
CD36-Associated (DBLd1-CIDRb1) (n = 20)	0.09	-0.24	0.17
CD36-Associated (DBLd1-CIDRgX) (n = 13)	0.28	-0.37	0.01
CD36-Associated (DBLd1-DBLbX) (n = 4)	-0.05	-0.41	-0.32
Miscellaneous (n = 20)	0.10	-0.46	0.00
var1 and var3 (n = 4)	0.18	-0.49	-0.11
Var2csa (n = 5)	0.06	-0.20	0.05
**Intracellular (n = 22)**	-0.02	-0.08	-0.10
ATSA (n = 6)	0.06	0.00	-0.03
ATSB (n = 16)	-0.07	-0.11	-0.12
**DBLalpha tag (n = 6)**	-0.31	-0.32	0.11
